# Survival predictors in elderly patients with acute respiratory distress syndrome: a prospective observational cohort study

**DOI:** 10.1038/s41598-018-31811-w

**Published:** 2018-09-07

**Authors:** Kuo-Chin Kao, Meng-Jer Hsieh, Shih-Wei Lin, Li-Pang Chuang, Chih-Hao Chang, Han-Chung Hu, Chiu-Hua Wang, Li-Fu Li, Chung-Chi Huang, Huang-Pin Wu

**Affiliations:** 1 Department of Thoracic Medicine, Chang Gung Memorial Hospital and Chang Gung University College of Medicine, Taoyuan, 333 Taiwan; 2 Department of Respiratory Therapy, Chang Gung University College of Medicine, Taoyuan, 333 Taiwan; 3 Department of Pulmonary and Critical Care Medicine, Chiayi Chang-Gung Memorial Hospital, Chiayi, 613 Taiwan; 4 Graduate Institute of Clinical Medical Sciences, School of Nursing, College of Medicine, Chang Gung University, Taoyuan, 259 Taiwan; 5 Division of Pulmonary, Critical Care and Sleep Medicine, Chang Gung Memorial Hospital and Chang Gung University College of Medicine, Keelung, 222 Taiwan

## Abstract

Acute respiratory distress syndrome (ARDS) has a high mortality rate in intensive care units (ICU). The elderly patients remain to be increased of ICU patients. The aim is to investigate the survival predictors of elderly patients with ARDS. We reported a prospective observational cohort research, including the patients with ARDS between October 2012 and May 2015. Demographic, comorbidities, severity, lung mechanics, laboratory data and survival outcomes were analyzed. A total of 463 patients with ARDS were ≥65 years old were enrolled and analyzed. Multivariate logistic regression analysis identified Charlson comorbidity index (CCI) [odds ratio (OR) 1.111, 95% CI 1.010–1.222, p = 0.031], Sequential Organ Failure Assessment (SOFA) score (OR 1.127, 95% CI 1.054–1.206, p < 0.001) and peak inspiratory pressure (PIP) (OR 1.061, 95% CI 1.024–1.099, p = 0.001) which were independently associated with hospital mortality. Regarding the subgroups patients as 65–74 years old, 75–84 years old and ≥85 years old, the baseline characteristics were not significant difference and the hospital mortality rates were also not significant difference. In conclusion, CCI, SOFA score and PIP were identified as survival predictors in elderly patient with ARDS. Assessing comorbidities with CCI is essential in predicting the survival for elderly patients with ARDS.

## Introduction

Some epidemiological studies have reported that acute respiratory distress syndrome (ARDS) accounts for 4% of all hospital admissions^1,2^, 10.4% of intensive care unit (ICU) admissions, and 23.4% of patients needed mechanical ventilation for more than 4 weeks^3^. Wang *et al*.^4^ reported that 15–20% of the patients with ARDS who survive will die by 1 year, mainly because of underlying comorbidities rather than pulmonary sequelae of ARDS. Furthermore, previous studies have reported that the mortality rate of ARDS among elderly patients may be as high as 69–80%^5,6^.

The number of elderly patients in the ICU continues to rise with the increasing age of the general population^7^. It has been estimated that 7% to 25% of patients in the ICU are 85 years old and older in developed countries^8,9^. Several studies have concluded that age is not a predictor of a poor prognosis for elderly patients admitted to an ICU, and that severity of chronic illness and premorbid functional status mainly decided the patients’ outcomes^9–11^. In addition, few studies have investigated the role of advanced age on the survival outcomes of patients with ARDS.

ARDS is a significant cause of morbidity and mortality in patients admitted to an ICU. Clinical trials on the management of ARDS usually exclude very old patients, however, these elderly patients will be admitted to an ICU more frequently and their management will be challenging. Therefore, the object of this study was to explore the survival predictors of elderly patients with ARDS. Understanding these factors may help intensivists when making decisions regarding the appropriate use of life support in this particular patient population.

## Material and Methods

### Study design and population

This prospective observational cohort research was conducted from October 2012 to May 2015 at Chang Gung Memorial Hospital, Linkou branch, a tertiary referral medical center in northern Taiwan. The hospital consists of 3,700 general ward beds and 278 adult ICU beds. All of the patients admitted to ICU needed invasive mechanical ventilation with available data on both PaO_2_/FiO_2_ ratio and chest X-ray were screened for eligibility via the Hospital Information System. This study was approved by the Institutional Review Board Ethics Committee of Chang Gung Memorial Hospital (CGMH IRB No. 102–1729B) and was carried out in accordance with relevant guidelines and regulations. All clinical investigations were conducted according to the principles expressed in the Declaration of Helsinki. The IRB approval exempted the study from informed consent due to the non-intervention and observational data collection nature.

### Data collection

We enrolled patients into this study if they met the criteria of the Berlin definition of ARDS^1^. Patients were excluded if they were younger than 18 years old, were referred from other hospitals, died within 48 hours, and had incomplete data. Demographics, baseline clinical characteristics and laboratory data were collected on enrollment. The following data were recorded on ICU admission: date of hospital and ICU admission, age, gender, predicted body weight, past underlying diseases history, risk factors and severity of ARDS on the day of diagnosis. The mechanical ventilator settings such as tidal volume, lowest PaO_2_/FiO_2_ ratio with the highest PEEP and peak inspiratory pressure (PIP) were recorded during mechanical ventilation when ARDS was recognized within the first 24 hours of ARDS diagnosis. The severity index were recorded within the first 24 hours of ARDS diagnosis including Charlson comorbidity index (CCI)^12^, Acute Physiology and Chronic Health Evaluation (APACHE) II score^13^, Sequential Organ Failure Assessment (SOFA) score^14^, and lung injury score^15^.

### Managements of ARDS

The general mechanical ventilation settings of the patients included a lung protective ventilation strategy using a low tidal volume of 4–8 mL/kg of the predicted body weight, and the PEEP setting guided by low PEEP - FiO_2_ table for volume-controlled or pressure-controlled ventilation. Oxygenation was monitored by SpO_2_ through pulse oximetry, and the FiO_2_ level was adjusted to maintain SpO_2_ at more than 90%. Hemodynamics and lung water were monitored if the clinical condition of the patient indicated the need using a PiCCO plus monitor (version 5.2.2; Pulsion Medical System AG, Muenchen, Germany).

### Statistical analysis

Data analysis was carried out by SPSS software version 22 (SPSS for Windows, SPSS Inc., Chicago, IL, USA). Student’s t test and ANOVA were used to compare the continuous variables. Categorical data were compared using the chi square test. The risk factor for hospital mortality was analyzed using univariate analysis, and the variables statistically significant (p < 0.05) were included for multivariate analysis by applying multiple logistic regressions based on backward elimination of data. Cumulative survival curves as a function of time were generated using the Kaplan-Meier approach and compared using the log-rank test. P value < 0.05 is considered to be statistically significant.

## Results

**Figure 1:**
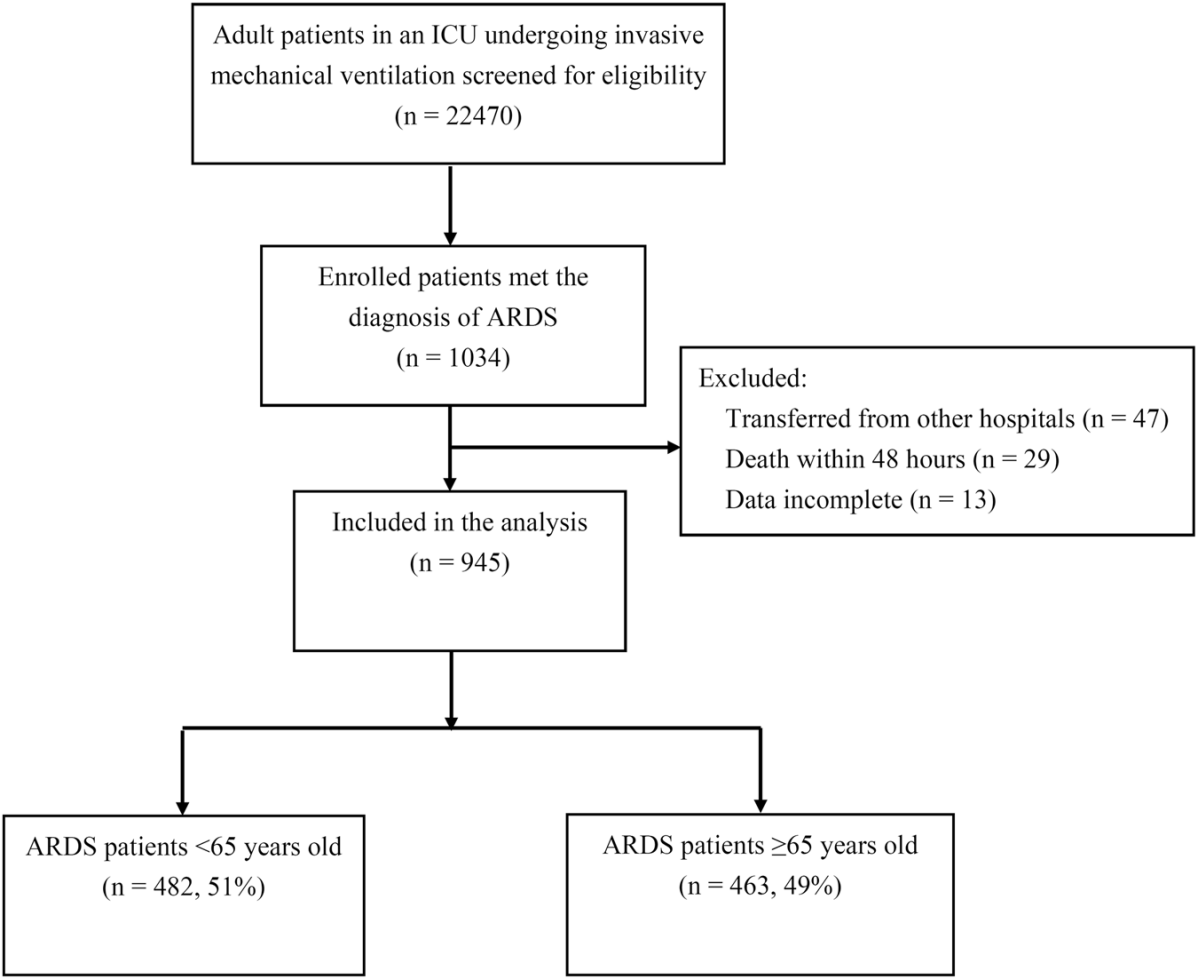
Flow chart of patient screening and enrollment for analysis. *ICU* intensive care, *ARDS* acute respiratory distress syndrome.

During the research period, 22,470 admitted adult patients with invasive mechanical ventilation were screened, of whom 1,034 (4.6%) met the criteria of ARDS (Fig. 1). The sources of patients included 9 medical ICUs, 5 post-surgical ICUs, 2 trauma ICUs, 1 burn ICU and emergency department. Eighty-nine patients were excluded, and the remaining 945 patients with ARDS were included for analysis.

The demographic and clinical characteristics of the included population are shown in Table 1. There were no significantly different in gender, CCI, lung injury score and mechanical ventilator settings between the younger (<65 years old) and older (≥65 years old) patients. The older patients had a lower body mass index and higher APACHE II and SOFA scores than the younger population. For the initial oxygenation, the older patients had a higher PaO_2_/FiO_2_ ratio (147.9 ± 77.3 vs. 134.3 ± 70.5 mmHg, p = 0.005) and less severe ARDS (34.1% vs. 40.2%, p = 0.041) than the younger patients. The hospital mortality rate was significantly higher in older patients than in younger patients (63.9% vs. 50.2%, p < 0.001). For the ARDS patients without co-morbidities (n = 186), the younger patients (n = 108) had lower hospital mortality rate than older patients (n = 78) (34.3% vs. 57.5%, p = 0.001).

**Table 1:** Demographics and baseline characteristics of the patients with ARDS by age groups.

Characteristics	Total patients	<65 years old	≥65 years old	p
	(n = 945)	(n = 482)	(n = 463)	
Age	62.2 ± 16.1	50.4 ± 11.1	76.5 ± 7.4	<0.001^*^
Gender (male/female)	653/292	345/137	308/155	0.093
BMI (kg/m^2^)	23.8 ± 4.5	24.3 ± 4.9	23.4 ± 4.1	0.002^*^
Charlson comorbidity index	2.6 ± 2.2	2.5 ± 2.3	2.6 ± 2.2	0.551
APACHE II score	23.2 ± 7.1	21.6 ± 7.0	24.9 ± 6.7	<0.001^*^
APACHE II score, without age	19.5 ± 6.9	21.6 ± 7.0	24.9 ± 6.7	0.258
SOFA score	9.7 ± 3.4	9.5 ± 3.2	10.0 ± 3.6	0.009^*^
Lung injury score	2.9 ± 0.5	2.9 ± 0.5	2.9 ± 0.5	0.834
Tidal volume/PBW (ml/kgw)	8.8 ± 2.1	8.9 ± 2.7	8.7 ± 2.9	0.803
PIP (cm H_2_O)	29.2 ± 5.7	29.6 ± 5.7	28.9 ± 5.8	0.093
PEEP (cm H_2_O)	9.9 ± 2.0	9.9 ± 2.2	9.8 ± 2.0	0.405
PaO_2_/FiO_2_ (mm Hg)	141.0 ± 74.2	134.3 ± 70.5	147.9 ± 77.3	0.005^*^
Severity of ARDS, n (%)				0.041^*^
Mild	213 (22.5)	94 (19.6)	119 (25.7)	
Moderate	380 (40.3)	194 (40.2)	186 (40.2)	
Severe	352 (37.2)	194 (40.2)	158 (34.1)	
Causes of ARDS				
Pneumonia	674	330	344	0.904
Sepsis	104	60	44	0.400
Aspiration	66	36	30	0.237
Post-surgery	44	25	19	0.409
Trauma	32	18	14	0.417
Others	25	13	12	0.479
Laboratory data				
Leukocytes (x 10^3^/mL)	13.6 ± 3.2	13.0 ± 1.1	14.2 ± 1.5	0.156
Hemoglobin (g/dL)	10.0 ± 2.4	10.1 ± 2.6	10.0 ± 2.1	0.533
Platelets (x 10^3^/µL)	156.9 ± 111.9	153.6 ± 120.9	160.4 ± 101.7	0.349
Albumin (g/dL)	2.6 ± 1.5	2.6 ± 0.6	2.7 ± 2.0	0.217
BUN (mg/dL)	33.5 ± 23.6	30.4 ± 23.3	36.6 ± 23.5	<0.001^*^
Creatinine (mg/dL)	1.8 ± 1.8	1.7 ± 1.8	1.9 ± 1.8	0.210
AST (U/L)	51.6 ± 30.9	56.9 ± 23.5	46.2 ± 28.2	<0.001^*^
ALT (U/L)	35.2 ± 28.9	39.4 ± 31.2	30.8 ± 25.5	<0.001^*^
Total Bilirubin (mg/dL)	1.0 ± 1.0	1.1 ± 1.1	1.0 ± 0.9	0.085
Na (mEq/L)	138.0 ± 9.5	138.6 ± 7.1	137.3 ± 11.4	0.787
K (mEq/L)	3.9 ± 0.9	3.9 ± 0.9	4.0 ± 0.9	0.104

ARDS: acute respiratory distress syndrome; BMI: body mass index; PBW: predicted body weight; APACHE: acute physical and chronic health evaluation; SOFA: sequential organ function assessment; PIP: peak inspiratory pressure; PEEP: positive end expiratory pressure; PaO_2_/FiO_2_: alveolar oxygen pressure/fraction of inspiratory oxygen.

All values are expressed as number of patients (%) or mean ± SD.

^*^Statistically significant difference between those aged <65 years old and those aged ≥65 years old.

The Table 2 compared the baseline characteristics of the older patients (≥65 years old) with ARDS between survivors and nonsurvivors. Regarding the risk factors of ARDS in these 463 older patients (≥65 years old), pneumonia was the most common (n = 354, 76.5%), followed by sepsis (n = 118, 25.5%), aspiration (n = 37, 8%), and others (n = 18, 4.2%). Of the 463 older patients with ARDS, the hospital survival rate was 36.1% (167/463). Univariate analysis showed that the CCI, APACHE II score, SOFA score, lung injury score and PIP were predictors of hospital mortality (Table 3). Multivariate logistic regression analysis revealed that CCI [odds ratio (OR) 1.111, 95% confidence interval (CI) 1.010–1.222, p = 0.031], SOFA score (OR 1.127, 95% CI 1.054–1.206, p < 0.001) and PIP (OR 1.061, 95% CI 1.024–1.099, p = 0.001) were significantly and independently associated with hospital mortality. Regression coefficients of these variables were used to calculate a natural logarithm of the odds (logit) of the probability of death (p), as follows: logit (p) = −2.5 + (0.11 × CCI) + (0.12 × SOFA score) + (0.06 × PIP).

**Table 2:** Demographics and baseline characteristics of the patients with ARDS aged ≥65 years old between survivors and nonsurvivors (n = 463).

Characteristics	Total patients	Survivors	Nonsurvivors	p
	(n = 463)	(n = 167)	(n = 296)	
Age	76.5 ± 7.4	76.5 ± 7.6	76.5 ± 7.2	0.968
Gender (male/female)	308/155	111/56	197/99	0.985
BMI (kg/m^2^)	23.6 ± 4.1	23.7 ± 4.1	23.2 ± 4.1	0.162
Charlson comorbidity index	2.6 ± 2.2	2.3 ± 2.1	2.9 ± 2.3	0.004^*^
APACHE II score	24.9 ± 6.7	23.7 ± 4.1	25.6 ± 6.7	0.005^*^
APACHE II score, without age	19.3 ± 6.7	18.1 ± 6.6	20.0 ± 6.7	0.447
SOFA score	9.5 ± 3.2	8.6 ± 2.8	9.9 ± 3.3	<0.001^*^
Lung injury score	2.9 ± 0.5	2.8 ± 0.5	2.9 ± 0.5	0.007^*^
Tidal volume/PBW (ml/kgw)	8.7 ± 2.9	8.9 ± 3.4	8.6 ± 2.6	0.357
Peak inspiratory pressure (cm H_2_O)	28.3 ± 5.8	27.7 ± 5.7	29.6 ± 5.7	<0.001^*^
PEEP (cm H_2_O)	9.8 ± 2.0	9.7 ± 1.9	9.9 ± 2.0	0.465
PaO_2_/FiO_2_ (mm Hg)	147.9 ± 77.4	152.4 ± 79.5	145.3 ± 76.1	0.397
Severity of ARDS, n (%)				0.536
Mild	119 (25.7)	43 (25.7)	76 (25.7)	
Moderate	186 (40.2)	72 (43.2)	114 (38.5)	
Severe	158 (34.1)	52 (31.1)	106 (35.8)	
Components of CCI, n (%)				
Myocardial infarct	13 (2.8)	5 (3.0)	8 (2.7)	0.860
Congestive heart failure	45 (9.7)	17 (10.2)	28(9.5)	0.802
Peripheral vascular disease	19 (4.1)	7 (4.2)	12(4.1)	0.943
Cerebrovascular disease	83 (17.9)	33 (19.8)	50(16.9)	0.440
Dementia	21 (4.5)	7 (4.2)	14(4.7)	0.789
Chronic lung disease	56 (12.1)	16 (9.6)	40(13.5)	0.213
Connective tissue disease	2 (0.4)	0 (0.0)	2(0.7)	0.287
Ulcer disease	48 (10.4)	13 (7.8)	35(11.8)	0.171
Mild liver disease	12 (2.6)	5 (3.0)	7 (2.4)	0.682
Diabetes without end organ damage	114 (31.1)	58 (34.7)	86 (29.1)	0.205
Hemiplegia or paraplegia	21 (4.5)	9 (5.4)	12 (4.1)	0.507
Moderate to severe renal disease	78 (16.8)	25 (15.0)	53 (17.9)	0.418
Diabetes with end organ damage	11 (2.4)	6 (3.4)	5 (1.7)	0.197
Any tumor without metastasis	89 (19.2)	25 (15.0)	64 (21.6)	0.081
Leukemia	1 (0.2)	0 (0.0)	1 (0.3)	0.452
Lymphoma	3 (0.6)	0 (0.0)	3 (1.0)	0.634
Moderate to severe liver disease	27 (5.8)	8 (4.8)	19 (6.4)	0.634
Metastatic solid tumor	48 (10.3)	10 (6.0)	38 (12.8)	0.123
AIDS	1 (0.2)	0 (0.0)	1 (0.3)	0.451

ARDS: acute respiratory distress syndrome; BMI: body mass index; APACHE: acute physical and chronic health evaluation; SOFA: sequential organ function assessment; PBW: predicted body weight; PEEP: positive end expiratory pressure; CCI: Charlson comorbidity index; PaO_2_/FiO_2_: alveolar oxygen pressure/fraction of inspiratory oxygen; AIDS: acquired immunodeficiency syndrome.

All values are expressed as number of patients (%) or mean ± SD.

^*^Statistically significant difference between survivors and nonsurvivors.

**Table 3:** Univariate and multivariate logistic regressions analyses of clinical variables associated with mortality in the patients with ARDS aged ≥65 years old (n = 463).

parameter	Beta coefficient	Standard error	Odds ratio (95% CI)	p
Univariate logistic regressions				
Age	0.00	0.01	1.00 (0.98–1.03)	0.968
BMI (kg/m^2^)	−0.03	0.02	0.97 (0.93–1.01)	0.967
Charlson comorbidity index	0.13	0.05	1.14 (1.04–1.24)	0.006^*^
APACHE II score	0.04	0.02	1.04 (1.01–1.07)	0.006^*^
APACHE II score, without age	0.04	0.01	1.04 (1.02–1.06)	<0.001^*^
SOFA score	0.14	0.03	1.15 (1.08–1.23)	<0.001^*^
Lung injury score	0.52	0.20	1.69 (1.15–2.48)	0.008^*^
Tidal volume/PBW	−0.03	0.03	0.97 (0.91–1.04)	0.361
Peak inspiratory pressure	0.06	0.01	1.06 (1.03–1.10)	0.001^*^
PEEP	0.04	0.05	1.04 (0.94–1.14)	0.465
PaO_2_/FiO_2_	0.00	0.00	1.00 (1.00–1.00)	0.341
Severity of ARDS				
Mild (reference)				
Moderate	−0.11	0.24	0.90 (0.56–1.44)	0.651
Severe	0.14	0.26	1.15 (0.70–1.90)	0.576
Multivariate logistic regressions				
Charlson comorbidity index	0.11	0.05	1.11 (1.01–1.22)	0.031^*^
SOFA score	0.12	0.03	1.18 (1.05–1.21)	<0.001^*^
Peak inspiratory pressure	0.06	0.02	1.06 (1.02–1.10)	0.001^*^
Constant	−2.5	0.61	0.082	<0.001^*^

ARDS: acute respiratory distress syndrome; BMI: body mass index; APACHE: acute physical and chronic health evaluation; SOFA: sequential organ function assessment; PBW: predicted body weight; PEEP: positive end expiratory pressure; PaO_2_/FiO_2_: alveolar oxygen pressure/fraction of inspiratory oxygen.

^*^Statistically significant difference.

Of these 463 older patients, 194 (41.9%) were 65–74 years old, 189 (40.8%) were 75–84 years old, and 80 (17.3%) were ≥85 years old. Demographic and clinical characteristics of these three age groups are compared in Table 4. There was no significant difference in gender, CCI, APACHE II, SOFA, lung injury score, mechanical ventilator settings and severity of ARDS among these three groups. For these older ARDS patients (≥65 years old), the ICU and hospital mortality rates were not significantly different in mild (n = 119), moderate (n = 186) and severe (n = 158) ARDS (43.7% vs. 47.8% vs 57%, respectively, p = 0.07; and 63.9% vs. 61.3% vs. 67.1%, respectively, p = 0.536).

**Table 4:** Demographics and baseline characteristics of the patients with ARDS aged ≥65 years old (n = 463).

Characteristics	65–74 years old (n = 194)	75–84 years old (n = 189)	≥85 years old (n = 80)	p
Age	69.2 ± 3.1	79.2 ± 2.8	87.5 ± 2.7	<0.001^*^
Gender (male/female)	124/70	126/63	58/22	0.391
BMI (kg/m^2^)	23.8 ± 4.1	23.4 ± 4.2	22.3 ± 3.4	0.025^*^
Charlson comorbidity index	2.8 ± 2.3	2.5 ± 2.2	2.5 ± 2.1	0.412
APACHE II score	24.5 ± 6.5	25.5 ± 6.7	24.6 ± 7.2	0.307
APACHE II score, without age	19.5 ± 6.5	19.5 ± 6.7	18.6 ± 7.2	0.556
SOFA score	9.6 ± 3.4	9.6 ± 3.0	8.9 ± 3.2	0.237
Lung injury score	2.9 ± 0.5	2.8 ± 0.5	2.8 ± 0.5	0.056
Tidal volume/PBW (ml/kgw)	8.7 ± 2.7	8.7 ± 2.8	8.7 ± 3.7	0.993
PIP (cm H_2_O)	29.6 ± 5.9	28.7 ± 5.7	27.8 ± 5.6	0.051
PEEP (cm H_2_O)	10.0 ± 2.1	9.7 ± 1.9	9.7 ± 1.9	0.291
PaO_2_/FiO_2_ (mm Hg)	143.6 ± 81.0	151.0 ± 74.0	151.1 ± 76.6	0.596
Severity of ARDS, n (%)				0.608
Mild	45 (23.2)	51 (27.0)	23 (28.7)	
Moderate	75 (38.7)	78 (41.3)	33 (41.3)	
Severe	74 (38.1)	60 (31.7)	24 (30.0)	
Laboratory data				
Leukocytes (x 10^3^/mL)	13.4 ± 1.8	14.5 ± 1.9	15.6 ± 1.9	0.545
Hemoglobin (g/dL)	10.1 ± 2.3	9.9 ± 2.0	10.0 ± 2.2	0.785
Platelets (x 10^3^/µL)	154.2 ± 94.3	163.8 ± 114.9	167.2 ± 84.8	0.528
Albumin (g/dL)	2.8 ± 1.9	2.7 ± 2.4	2.5 ± 0.6	0.777
BUN (mg/dL)	35.9 ± 25.0	35.9 ± 22.8	39.6 ± 21.6	0.478
Creatinine (mg/dL)	1.9 ± 2.0	1.9 ± 1.7	1.8 ± 1.4	0.874
AST (U/L)	49.1 ± 28.3	46.0 ± 30.4	39.2 ± 21.5	0.122
ALT (U/L)	32.8 ± 25.4	30.5 ± 26.7	27.0 ± 22.1	0.325
Total Bilirubin (mg/dL)	1.0 ± 1.0	1.0 ± 0.8	0.8 ± 0.8	0.211
Na (mEq/L)	136.9 ± 12.1	137.2 ± 12.0	139.0 ± 8.0	0.304
K (mEq/L)	4.0 ± 0.9	4.0 ± 0.8	4.1 ± 0.9	0.114

ARDS: acute respiratory distress syndrome; BMI: body mass index; PBW: predicted body weight; APACHE: acute physical and chronic health evaluation; SOFA: sequential organ function assessment; PIP: peak inspiratory pressure; PEEP: positive end expiratory pressure; PaO_2_/FiO_2_: alveolar oxygen pressure/fraction of inspiratory oxygen.

All values are expressed as number of patients (%) or mean ± SD.

^*^Statistically significant difference between those aged 65–74 years old and those aged ≥85 years old.

The ≥85 years old group had a significantly lower body mass index than the 65–74 years group (22.3 ± 3.4 vs. 23.8 ± 4.1, p = 0.025). There was no significant difference in ICU or hospital mortality rates among the three groups (45% vs. 48.7% vs. 53.1%, respectively, p = 0.433; and 60% vs. 65.6% vs. 63.9%, respectively, p = 0.682), and no significantly different in days of mechanical ventilation among the three groups (19.1 ± 14.6 days, 20.5 ± 15.7 days, and 21.0 ± 15.4 days, respectively, p = 0.583). The lengths of stay in the ICU and hospital were not significantly different among the three groups (24.0 ± 18.8 days vs. 25.9 ± 21.0 days vs. 26.4 ± 20.6 days, respectively, p = 0.565; and 34.7 ± 29.2 days vs. 38.2 ± 31.8 days vs. 35.7 ± 23.4 days, respectively, p = 0.459). The leading causes of death of the older patients with ARDS were multiple organ failure (n = 203), followed by septic shock (n = 46) and refractory hypoxemia (n = 20). Between the three groups, these three leading causes of death were not significant difference (68% vs. 72% vs. 69%, p = 0.81; 17% vs. 15% vs. 16%, p = 0.846; 7% vs. 6% vs. 8%, p = 0.947). For the older patients without co-morbidities (n = 78), the hospital mortality rates were not significantly different between 65–74 years old (n = 32), 75–84 years old (n = 32), and ≥85 years old (n = 14). (56.2% vs. 56.2 vs. 64.3%, respectively, p = 0.859).

**Figure 2:**
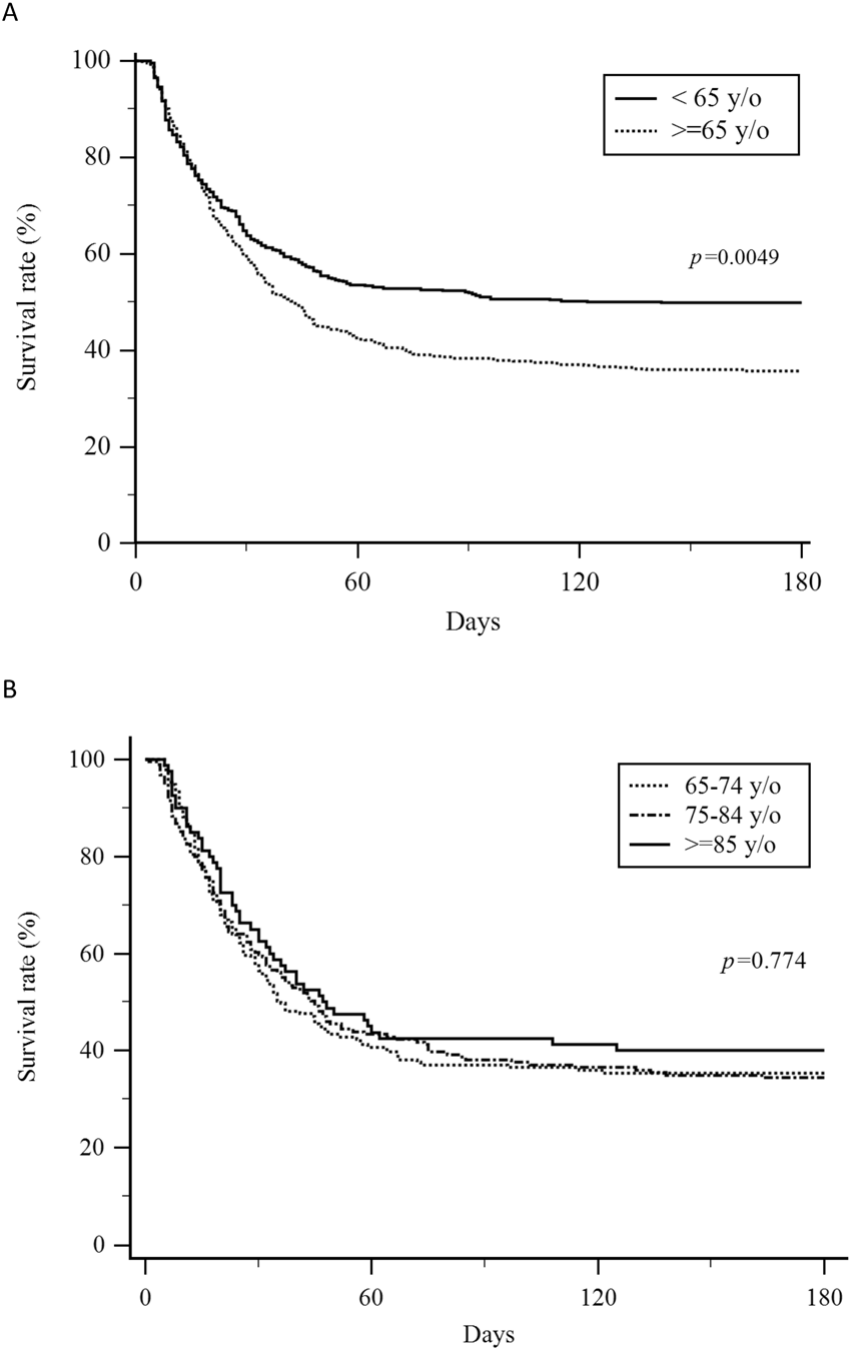
Kaplan-Meier survival curves of patients with acute respiratory distress syndrome in different age groups. (**A**) The patients <65 years old had a significantly higher survival rate than those ≥65 years old (p = 0.0049). (**B**) The survival rate was not significantly different among those aged 65–74 years, 75–84 years old and ≥85 years old groups (p = 0.774).

Kaplan-Meier survival curves for hospital survival in the different age groups are shown in Fig. 2. The younger patients (<65 years old) had a significantly higher survival rate than the older patients (≥65 years old) (p = 0.0049). However, the survival rate was not significantly different among the 65–74, 75–84 and ≥85 years old groups (p = 0.774).

## Discussion

The main results of this prospective observational cohort study revealed that the older (≥65 years old) patients with ARDS had a lower survival rate than the younger (<65 years old) patients with ARDS. For the elder ARDS patients, the CCI, SOFA score and PIP were significantly and independently associated with hospital mortality. However there was no significant difference in ICU or hospital survival rates among the 65–74 years old, 75–84 years old and ≥85 years old groups.

The Berlin definition classifies the severity of ARDS by the PaO_2_/FiO_2_ ratio, and mild, moderate, and severe ARDS are associated with increased mortality (27%, 32% and 45%, respectively)^1^. Recent reports have shown that stratification of severity of ARDS based on baseline value of PaO_2_/FiO_2_ did not completely correlate with mortality^16–18^. Often as much as 50% of patients classified as having moderate or severe ARDS respond quickly to routine ventilator and oxygenation measures that they do not meet the criteria for moderate/severe ARDS at 24 hours after diagnosis^17^. A 9 - point score based on age, PaO_2_/FiO_2_, and plateau pressure was proposed to predict mortality in patients with ARDS^19^. Compared to patients with ARDS who were younger than 47 years old, those 47–66 years and >66 years old had significantly higher hospital mortality rates (27.5% vs. 44.4% vs. 66.0%, respectively; p < 0.001)^19^. Increasing age is a known risk factor for death in patients with ARDS, and older patients have a higher risk of mortality than younger patients^20,21^. However, little is known about the risk of mortality for patients with ARDS who are older than 65 years. In this study, we found that the major determinants of mortality were underlying disease (e.g. CCI), organ function (e.g. SOFA score) and pulmonary condition (e.g. PIP), but not age. Therefore, the impact of age on mortality in patients with ARDS seems to be limited, especially in elderly patients.

A study on patients with ARDS found that patients with serious comorbidities had a mortality rate three times higher than patients without serious comorbidities^22^. The CCI is an index of multiple comorbidities including 22 items which was initially developed in a cohort of 559 internal medicine patients to predict 1 year mortality^16^. In lung cancer patients, the CCI has been shown to be a prognostic predictor^23^, and several studies have reported that the CCI can predict survival and physiological outcomes in patients with ARDS^24–26^. In this study on elderly patients with ARDS, we found that the CCI was significantly positively correlated with survival outcomes (OR 1.11, p = 0.031). Therefore, we suggest assessing comorbidities using the CCI to predict survival in elderly patients with ARDS in addition to age.

The SOFA score involves organ dysfunction across six vital organs and it has been shown to be associated with more severe disease and a higher risk of death^14^. Only about 20% of patients with ARDS die from refractory hypoxemia, and approximately 80% of all deaths are caused by multiple organ dysfunction syndromes^27,28^. The SOFA score has been used for patients with ARDS to evaluate organ dysfunction as a surrogate marker of mortality^29^. In terms of liver failure, patients with ARDS and cirrhosis have been reported to have a significantly higher mortality rate (62%) than patients without cirrhosis (43%) (p = 0.02)^30^. An observational study of patients with indirect ARDS found that age, lung injury score, and number of non-pulmonary organ failures (OR 1.67, p = 0.01) were independent risk factors for hospital mortality^31^. The LUNG SAFE study of patients with ARDS found that a higher non-pulmonary SOFA score was associated with poorer outcomes (OR 1.12, p < 0.001)^32^. In our study on elderly patients with ARDS, SOFA score was significantly correlated with hospital mortality (OR 1.18, p < 0.001). The prognosis for elderly patients with ARDS therefore appears to be related to extra-pulmonary organ dysfunction rather than pulmonary dysfunction alone.

A study including 3562 patients with ARDS in nine randomized controlled trials concluded that driving pressure as an index of pulmonary mechanics of the respiratory system was the strongest predictor of mortality^33^. Another study on 56 patients with ARDS reported that treatment strategies leading to decreased transpulmonary driving pressure at 24 hours may be associated with an improved 28 - day mortality rate^34^. In addition to driving pressure, plateau pressure has also been reported to be a predictor of mortality in patients with ARDS^19,32^. A prospective, descriptive, and validation study reported that the hospital mortality rates of patients with ARDS with a plateau pressure >30 cm H_2_O and <27 cm H_2_O were 64.0% and 28.7%, respectively (relative risk 2.2, p < 0.001)^19^. Peak inspiratory pressure, which is an easily measurable parameter of lung mechanics was also associated with hospital mortality in the LUNG SAFE study (OR 1.02, p = 0.002)^32^. For our elderly patients with ARDS, we found that PIP was significantly correlated with hospital mortality (OR 1.068, p = 0.001). In theory, the PIP is different from plateau pressure. However, the peak airway has a good collinearity with plateau pressure especially when patients were to be deep sedated or paralyzed in early stage of ARDS. Due to its convenience and feasibility, PIP may be useful as a prognostic index in real world patient care.

There are several limitations to this study. First, this study was conducted at one referral medical center, and our results may not be generalizable to patients in community hospitals or other models of intensive care. Nevertheless, the number of enrolled elderly patients with ARDS was reasonably high, and thus we believe our findings are of value. Second, few studies have investigated elderly patients with ARDS, and these studies have mostly focused on critically ill patients as a whole. We chose 65 years of age as a cutoff value mainly because previous studies on critically ill patients have used this cutoff to define “elderly” patients. We further classified the elderly patients into three arbitrary age groups of 65–74, 75–84 and ≥85 years old without considering morbidities, functional status and other disabilities, and this may have affected the outcomes. Third, different health care systems in different countries will have different policies for intensive care for critically ill elderly patients. Finally, it is possible to have a selection bias from the patients’ collection. Some patients were possibly rejected to be admitted in ICU because of underlying comorbidity by the physician in charge.

In conclusion, CCI, SOFA score and PIP were predictors of hospital survival in elderly patients with ARDS. The risk of mortality in the elderly patients ARDS was associated with the degree of lung injury and also with the underlying disease and presence of other organ dysfunction. When making decisions regarding life-sustaining therapy for elderly patients with ARDS, both comorbidities and advanced age should be taken into consideration.
